# Prevalence of genetic polymorphisms in the promoter region of the alpha-1 antitrypsin (*SERPINA1*) gene in chronic liver disease: a case control study

**DOI:** 10.1186/1471-230X-10-22

**Published:** 2010-02-20

**Authors:** Karin F Kok, René H te Morsche, Martijn GH van Oijen, Joost PH Drenth

**Affiliations:** 1 Department of Gastroenterology and Hepatology, Radboud University Nijmegen Medical Centre, Nijmegen, the Netherlands

## Abstract

**Background:**

Alpha-1 antitrypsin (A1AT) deficiency, caused by the Z allele *(p.E342K) *and S allele *(p.E264V) *in the *SERPINA1 *gene, can induce liver and pulmonary disease. Different mechanisms appear to be responsible for the pathogenesis of these divergent disease expressions. The *c.-1973T *>*C *polymorphism located in the *SERPINA1 *promoter region is found more frequent in A1AT deficiency patients with liver disease compared to patients with pulmonary disease, but data are lacking regarding contribution to the development of liver diseases caused by other aetiologies.

**Aim:**

To study the prevalence of *c.-1973T *>*C*, Z allele and S allele in a cohort of patients with liver disease of various aetiologies compared with healthy controls and to evaluate its effect on disease progression.

**Methods:**

A total of 297 patients with liver disease from various aetiologies and 297 age and gender matched healthy controls were included. The *c.-1973T *>*C *polymorphism and Z and S alleles of the *SERPINA1 *gene were analyzed by real-time PCR.

**Results:**

*c.-1973T *>*C *was similarly distributed between patients with liver disease of various origins and healthy controls. Furthermore, the distribution of *c.-1973T *>*C *was independent from aetiology subgroup. In patients with liver disease mean ages at of onset of liver disease were 44.4, 42.3 and 40.7 years for the *c.-1973 *T/T, T/C and C/C genotype respectively (NS). S allele heterozygosity was increased in patients with drug induced liver injury (DILI), (OR 4.3; 95%CI 1.1-17.2).

**Conclusion:**

In our study, *c.-1973T *>*C *polymorphism was not a risk factor for liver disease of various aetiologies. In addition, S allele heterozygosity might contribute to the development of DILI.

## Background

Alpha-1 antitrypsin (A1AT) deficiency is a hereditary disease and can induce end-organ damage caused by defective A1AT protein processing. Liver disease in A1AT deficiency is caused by hepatic accumulation of the A1AT protein and pulmonary disease is induced by an impaired protection against neutrophil elastase due to decreased serum A1AT[1,2]. The A1AT protein is encoded by the protease inhibitor (Pi) locus located on chromosome 14q32.1 (*SERPINA1 *gene). In Western Europe, A1AT deficiency most commonly results from presence of 2 genetic variants *p.E342K *(denoted as Z allele) and *p.E264V *(commonly referred to as the S allele)[3]. Liver disease in A1AT deficiency has a bimodal presentation affecting children in neonatal life [4] and, less commonly, adults in late middle life[5].

It is unclear why A1AT deficiency leads to liver disease in some patients and lung disease in others. It appears that environmental factors are in part responsible for this difference. Pulmonary disease develops preferably in homozygous Pi ZZ persons who are tobacco smokers or are exposed to airway irritants[6]. Indeed, smoking is an established risk factor for lung disease as A1AT deficient smokers will develop emphysema at considerable younger age, while non-smokers are at risk for liver disease developing later in life[7]. Some 32-37% of A1AT deficient non-smoking patients will die as a result of A1AT deficiency induced liver disease[8].

Apart from environmental factors there is some evidence that genetic factors modify the risk for A1AT deficiency related end-organ damage. For example, 72% of siblings of probands with A1AT deficiency related liver disease suffered from liver disease, which was concordant for severity in 29%, while 28% had no liver involvement[9]. This suggests presence of genetic modifiers. Indeed a recent study identified a novel single nucleotide polymorphism (SNP) *g.126076T *>*C (c.-1973T *>*C; *rs8004738) in the promoter region of *SERPINA1*. It appeared that the SNP was enriched (15.5%) in a cohort of A1AT Pi ZZ homozygotes with liver disease relative to those with pulmonary disease (6.5%)[10].

As a result of the above-mentioned observations we hypothesized that *c.-1973T *>*C *polymorphism affects susceptibility for the progression of liver disease in patients with liver disease of various aetiologies. Therefore we investigated the association of *c.-1973T *>*C*, *p.E342K *(Z allele) and *p.E264V *(S allele) polymorphisms in a cohort of patients with liver disease of various aetiologies compared with healthy controls and evaluated its consequence on course of disease.

## Methods

### Patients

We recruited patients with various liver disorders, visiting the outpatient clinic of the Department of Gastroenterology and Hepatology of the Radboud University Nijmegen Medical Center. In addition, age and gender matched persons who were unrelated to our patients served as healthy controls. In the patient population, clinical and demographic data including age, sex, age at first presentation of liver disease, aetiology of liver disease and presence or absence of cirrhosis were obtained. The absence of liver disease in our control population was established on the basis of self-reporting and none of the patients used any medication. Whole blood samples were stored at -20°C. Altogether the study population comprised 297 patients with various aetiologies of liver disease and 297 controls. Clinical and demographic data are given in Table 1. The study was approved by the local ethical committee (Medical Ethical Committee of the Radboud University Nijmegen Medical Center) and all subjects gave their informed consent.

**Table 1:** Baseline characteristics of patients and controls.

	*Patients*	*Controls*	*p-value*
	*(n = 297)*	*(n = 297)*	
*Male (%)*	168 (57)	163 (55)	0.74
*Mean age (range)*	51.4 yrs (19-85)	51.5 yrs (20-85)	0.94
*Mean age at onset liver disease (range)*	43 yrs (7-82)		
*Cirrhosis (%)*	69 (23)		
*Cause of liver disease (%)*			
-HCV	129 (43)		
-AIH/PBC/PCS	53 (18)		
-HBV	50 (17)		
-Alcoholic liver disease	21 (7)		
-NASH/metabolic	16 (5)		
-Cryptogenic	13 (4)		
-Drug induced liver injury	11 (4)		
-Vascular	4 (1)		

HCV = hepatitis C virus, AIH/PSC/PBC = autoimmune hepatitis/primary biliary cirrhosis/primary sclerosing cirrhosis, HBV = hepatitis B virus, NASH = non-alcoholic steato hepatitis

### Laboratory

DNA was isolated from peripheral blood using the High Pure PCR Template preparation kit (Roche, Mannheim, Germany). The *c.-1973T *>*C *(rs8004738), *p.E342K *(Z allele; c.1024G > A; rs28929474) and *p.E264V *(S allele; c.791A > T; rs17580) polymorphisms of the *SERPINA1 *gene were analyzed by real-time polymerase chain reaction (PCR) using a dual-color, allele-specific discrimination assay with fluorescent labelled probes on the iCycler iQ Multicolour real-time detection system (Bio-Rad Laboratories Inc, Hercules, CA, USA). Primer and probe sequences (Sigma, St Louis, MO, USA) used for PCR and real time detection are listed in additional file 1. All of the genotyping results in the control population were in the Hardy-Weinberg equilibrium. Haplotypes and diplotypes were determined using the Partition-Ligation-Expectation-Maximization (PLEM) 1.0 software [11].

### Statistical analysis

Baseline characteristics, differences in allele frequency, diplotypes and haplotypes were analyzed using student t-tests, Pearson chi-squared test and Fisher's exact test when appropriate. Odds Ratio's (OR) were calculated for the association between the studied polymorphisms and the presence of liver disease. In addition we calculated OR's for the association between the 3 polymorphisms and all different subgroup aetiologies. We analyzed differences in age at onset of liver disease between the 3 different *c.-1973 *genotypes using ANOVA. All statistical analyses were performed using GraphPad Prism Version 4.02 (GraphPad Software Inc., San Diego, CA, USA). A two-sided p-value of < 0.05 was considered statistically significant. Post-hoc power calculation showed the study being adequate powered (88%) to negate our hypothesis that *c.-1973T *>*C *is associated with liver disease of various aetiologies (α = 0.05, difference in group proportions 10% (20% and 30%)). Power calculations were performed using nQuery Advisor 4.0 software (Statistical Solutions Ltd, Cork, Ireland). Linkage disequilibrium (LD) values were performed with Haploview 4.0 software.

## Results

### c.-1973T > C polymorphism

**Figure 1:**
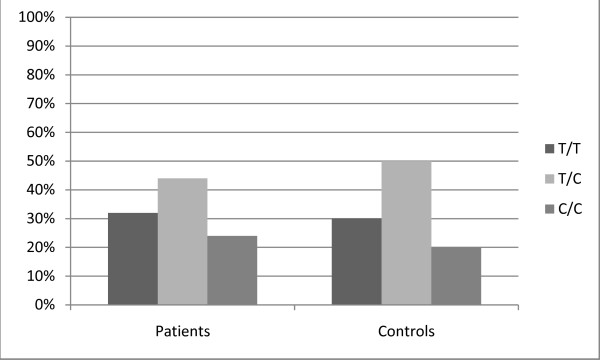
**Distribution of the *c.-1973T *>*C *genotypes in 297 patients with liver disease of various aetiologies and 297 controls**.

**Figure 2:**
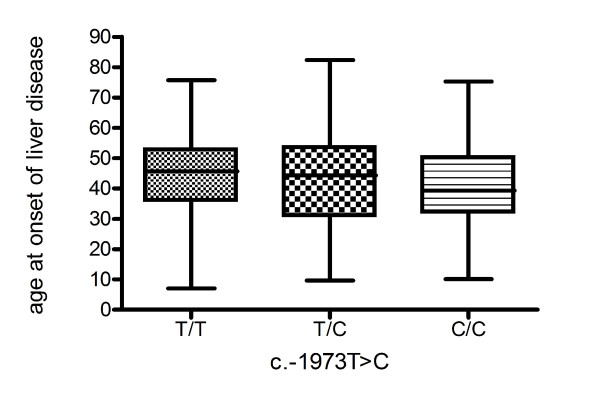
**Age at onset of liver disease in 297 patients arranged to the different *c.-1973T *>*C *genotypes**.

The *c.-1973T *>*C *polymorphism distribution in 297 patients with liver disease due to various aetiologies was in line with 297 healthy controls. (*c.-1973*: T/T 32%, T/C 44% and C/C 24% in patients and T/T 30%, T/C 50% and C/C 20% in controls). (Figure 1) We found no association of the *c.-1973T *>*C *polymorphism with any of the distinct liver diseases investigated. Next, we observed that mean age at onset of liver disease was non-significantly lower in patients with the *c.-1973 *C/C allele, as mean ages of onset of liver disease were 44.4 (T/T), 42.3 (T/C) and 40.7 (C/C) years. (Figure 2) Further, possession of *c.-1973T *>*C *polymorphism had no influence on the presence of cirrhosis.

### Z (p.E342K) allele and S (p.E264V) allele heterozygosity

We observed a similar Z allele and S allele heterozygosity rate in patients and controls (Z allele: 3.0% and 4.7%; S allele: 6.7% and 8.0%). The distribution of Z allele heterozygosity was similar among all liver diseases of various aetiologies. In contrast, S allele heterozygosity was more frequently present in drug induced liver injury (DILI) compared with healthy controls (11 patients, 27% vs 8%; OR 4.27; 95%CI 1.06-17.15). A total of 8 wildtype patients developed DILI likely due to clavulanic acid (n = 3), azatioprine, sulfasalazine, pantoprazole, methotrexate and quetiapine; addionally, the S allele heterozygotes had DILI caused by anaesthetic compounds (isoflurane/nestonal), clavulanic acid and celecoxib. Lastly, age at onset of liver disease was independent of Z or S allele heterozygosity as mean ages were 48.3 (Z allele), 43.2 (S allele) and 42.4 (wildtypes) years.

### Haplotyping/diplotyping

**Figure 3:**
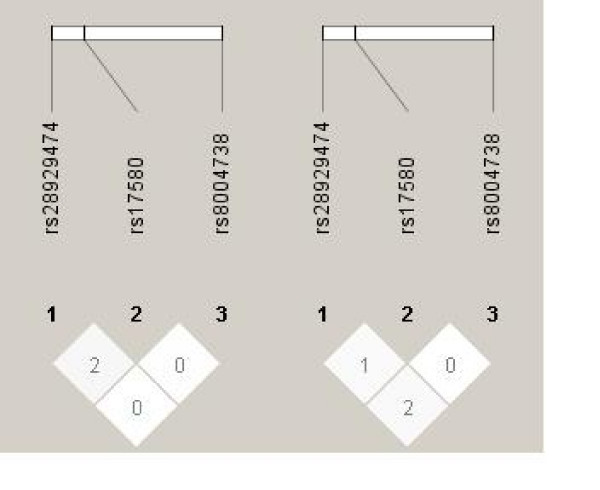
**Linkage disequilibrium (LD) plot across the *SERPINA1 *gene**. Patients. Controls. The box at the top indicates the *SERPINA1 *gene with the 3 investigated SNP's (1 = *p.E342K*, 2 = *p.E264V *and 3 = *c.-1973T *>*C*). The LD plot is based on the measurement of R^2 ^(values × 0.01). Each diamond indicates the pair wise magnitude of LD. (LD: linkage disequilibrium is the non-random association of alleles at two or more loci. LD describes a situation in which some combinations of alleles or genetic markers occur more or less frequently in a population than would be expected from a random formation of haplotypes from alleles based on their frequencies.)

Based on the 3 polymorphisms tested, haplotype and diplotype analysis were performed. A total of 11 diplotypes could be distinguished. We could not detect differences in diplotypes between patients with liver disease of various etiologies and controls. Only the CTG haplotype was more frequent found in patients with DILI, according to the above-described association between S allele heterozygosity and DILI. (Table 2) Finally, linkage disequilibrium (LD) between the 3 *SERPINA1 *alleles was absent. This was true for patients as well as controls. (Figure 3)

**Table 2:** Diplotypes in patients with liver disease of various aetiology and healthy controls

Diplotype	Controls (%)	Patients (%)
	n = 297	n = 297
**C**AG/TAG	130 (43.8)	112 (37.7)
TAG/TAG	78 (26.3)	93 (31.3)
**C**AG/**C**AG	51 (17.2)	63 (21.2)
**CT**G/TAG	11 (3.7)	12 (4.0)
**C**AG/**CT**G	8 (2.7)	7 (2.4)
TAG/TA**A**	8 (2.7)	2 (0.7)
**C**AG/TA**A**	5 (1.7)	6 (2.0)
TAG/T**T**G	4 (1.3)	1 (0.3)
**CT**G/TA**A**	1 (0.3)	
**CT**G/**CT**G	1 (0.3)	
**CA**G/**C**A**A**		1 (0.3)
		

Order of alleles: *c.-1973T *>*C*- c.791A > T - c.1024G > A/*c.-1973T *>*C*- c.791A > T - c.1024G > A (mutations in **bold**)

## Discussion

We show that the distribution of *SERPINA1 c.-1973T *>*C *in patients with liver disease from various aetiologies is similar compared to healthy controls. The genotype frequencies in the patient and control groups were in line with those from the HapMap database (TT 30%, T/C 45% and C/C 25%) [12]. Moreover, we found that the distribution of *c.-1973T *>*C *is independent from aetiology of the liver disease. We also examined whether the *c.-1973T *>*C *polymorphism affected age at onset of liver disease. We found a non-significant lower age at onset in patients with the *c.-1973 *C/C genotype compared to other *c.-1973 *genotypes. In addition, we could not demonstrate an association of *c.-1973T *>*C *on severity of liver disease e.g. cirrhosis. We also investigated other SERPINA1 variants and found that there was no increased prevalence of Z and S alleles in patients with liver disease of various aetiologies compared with healthy controls, even though we observed an enrichment of S allele heterozygosity in patients with DILI.

Other investigators have studied the presence of *c.-1973T *>*C *and A1AT deficiency and chronic obstructive pulmonary disease (COPD). Chappell et al. reported an enrichment of *c.-1973T *>*C *in homozygous Pi ZZ neonates with hepatitis (15.5%) compared to homozygous Pi ZZ controls (adults with COPD and unaffected subjects) (6.5%)[10]. Since our study population consisted for a large extent of patients and controls with the Pi MM genotype (wildtypes) and hardly any subjects with Z and S allele heterozygosity, we cannot compare our data with the results of the above mentioned study. Another study showed a decreased prevalence of *c.-1973T *>*C *in patients with COPD (48.7%) compared to controls (52.2%), suggesting a protective effect against COPD[13]. It might be possible that *c.-1973T *>*C *influences the genesis of liver disease in childhood, in line with a recently published report implicating that a variant of the endoplasmic reticulum mannosidase I (ERManI) gene is associated with an early onset of end-stage liver disease in patients with homozygous (Pi ZZ) A1AT deficiency[14].

Our data were in contrast with other reports as Z and S allele heterozygosity were previously associated with (end-stage) liver disease due to HCV, alcoholic liver disease and cryptogenic cirrhosis [15-19] and not with DILI. We found a higher frequency of S allele heterozygosity in DILI patients. Indeed, experimental evidence supports a relation between A1AT deficiency and DILI as administration of indomethacin in a homozygous Pi ZZ mouse model leads to increased hepatic injury [20] and a case report described prochlorperazine induced liver injury in a homozygous (Pi ZZ) A1AT deficient patient [21].

We could not demonstrate an association between *c.-1973T *>*C *and the presence of cirrhosis. There have been several genetic case control studies that have attempted to detect associations between genetic variations and liver fibrosis. For example, the combination of angiotensinogen (ATG) gene variant *c.1-44 *and transforming growth factor beta (TGFβ1) p.R25P is associated with advanced hepatic fibrosis in obese patients with non alcoholic fatty liver disease [22] but not in patients with other chronic liver diseases[23]. Lastly, matrix metalloproteinase (MMP)-7(Asp-137) confers risk of liver cirrhosis[24].

Our study comes with limitations. Our cohort could have suffered from selection bias due to patient recruitment in a tertiary referral centre. The strength of our study is the sufficiently power to negate a 10% difference in prevalence of the *c.-1973T *>*C *polymorphism between groups. However, the study lacks power to demonstrate smaller differences and to perform a thorough subgroup analysis. Further research regarding *c.-1973T *>*C *should include homozygous Pi ZZ adults with liver disease to evaluate whether *c.-1973T *>*C *is a risk factor for a hepatic expression of A1AT deficiency.

## Conclusion

We demonstrated that, in our study, *c.-1973T *>*C *polymorphism was not associated with liver disease of various aetiologies. In addition, S allele heterozygosity might be a risk factor for the genesis of DILI.

## Competing interests

The authors declare that they have no competing interests.

## Authors' contributions

KK carried out the molecular genetic studies, participated in the design of the study, participated in the statistical analysis and drafted the manuscript. RM generated the sequence alignment, constructed the diplo- and haplotypes and participated in the molecular genetic studies. MO participated in the statistical analysis and read previous drafts of the manuscript. JD, the principle investigator participated in the design of the study and coordinated the study. All authors read and approved the final version.

## Pre-publication history

The pre-publication history for this paper can be accessed here:

http://www.biomedcentral.com/1471-230X/10/22/prepub

## Supplementary Material

Additional file 1 Overview of primers and probes used for real time PCR reactions. Click here for file
