# Supplementary material for: Prevalence of genetic polymorphisms in the promoter region of the alpha-1 antitrypsin (SERPINA1) gene in chronic liver disease: a case control study
Source: BMC Gastroenterol. 2010 Feb 20;10:22. doi: 10.1186/1471-230X-10-22 (PMC2843604; doi:10.1186/1471-230X-10-22)
Supplement: Additional file 1 — Overview of primers and probes used for real time PCR reactions. [file 1471-230X-10-22-S1.DOCX]

| Z allele *(p.E342K)* | Forward primer | AAAACATGGCCCCAGCAG |
| --- | --- | --- |
|  | Reverse primer | AGCCTTACAAGTGTCTCTG |
|  | Probe wildtype | Fam-TCAGTCCCTTTCTCGTCGATGGTCAG-BHQ1 |
|  | Probe mutation | Hex-TCAGTCCCTTTCTTGTCGATGGTCAG-BHQ1 |
| S allele  *(p.E264V)* | Forward primer | AACATGGCTAAGAGGTGTGG |
|  | Reverse primer | ATCTTCTTCCTGCCTGATGAG |
|  | Probe wildtype | Fam-CTACAGCACCTGGAAAATGAACTCACCC-BHQ1 |
|  | Probe mutation | Hex-CTACAGCACCTGGTAAATGAACTCACCC-BHQ1 |
| *c.-1973T>C* | Forward primer | AGGCTGGCAGGAGGTTGC |
|  | Reverse primer | GAGCTGAACCAAGAAGGAGGAG |
|  | Probe C | Fam-TGGCAGCAGCAGCGGCTAGGCC-BHQ1 |
|  | Probe T | Hex-TGGCAGCAGCAGCAGCTAGGCC-BHQ1 |
